# Two shikimate dehydrogenases play an essential role in the biosynthesis of galloylated catechins in tea plants

**DOI:** 10.1093/hr/uhae356

**Published:** 2024-12-23

**Authors:** Liubin Wang, Yongxin Wang, Yueqi Wang, Liyun Wu, Mengdi He, Zhuozhuo Mao, Guanhua Liu, Kang Wei, Liyuan Wang

**Affiliations:** Key Laboratory of Biology, Genetics and Breeding of Special Economic Animals and Plants, Ministry of Agriculture and Rural Affairs, National Center for Tea Improvement, Tea Research Institute, Chinese Academy of Agricultural Sciences (TRICAAS), 9 Meiling South Road, Hangzhou 310008, China; Key Laboratory of Biology, Genetics and Breeding of Special Economic Animals and Plants, Ministry of Agriculture and Rural Affairs, National Center for Tea Improvement, Tea Research Institute, Chinese Academy of Agricultural Sciences (TRICAAS), 9 Meiling South Road, Hangzhou 310008, China; Key Laboratory of Biology, Genetics and Breeding of Special Economic Animals and Plants, Ministry of Agriculture and Rural Affairs, National Center for Tea Improvement, Tea Research Institute, Chinese Academy of Agricultural Sciences (TRICAAS), 9 Meiling South Road, Hangzhou 310008, China; Key Laboratory of Biology, Genetics and Breeding of Special Economic Animals and Plants, Ministry of Agriculture and Rural Affairs, National Center for Tea Improvement, Tea Research Institute, Chinese Academy of Agricultural Sciences (TRICAAS), 9 Meiling South Road, Hangzhou 310008, China; Key Laboratory of Biology, Genetics and Breeding of Special Economic Animals and Plants, Ministry of Agriculture and Rural Affairs, National Center for Tea Improvement, Tea Research Institute, Chinese Academy of Agricultural Sciences (TRICAAS), 9 Meiling South Road, Hangzhou 310008, China; Key Laboratory of Biology, Genetics and Breeding of Special Economic Animals and Plants, Ministry of Agriculture and Rural Affairs, National Center for Tea Improvement, Tea Research Institute, Chinese Academy of Agricultural Sciences (TRICAAS), 9 Meiling South Road, Hangzhou 310008, China; Key Laboratory of Biology, Genetics and Breeding of Special Economic Animals and Plants, Ministry of Agriculture and Rural Affairs, National Center for Tea Improvement, Tea Research Institute, Chinese Academy of Agricultural Sciences (TRICAAS), 9 Meiling South Road, Hangzhou 310008, China; Key Laboratory of Biology, Genetics and Breeding of Special Economic Animals and Plants, Ministry of Agriculture and Rural Affairs, National Center for Tea Improvement, Tea Research Institute, Chinese Academy of Agricultural Sciences (TRICAAS), 9 Meiling South Road, Hangzhou 310008, China; Key Laboratory of Biology, Genetics and Breeding of Special Economic Animals and Plants, Ministry of Agriculture and Rural Affairs, National Center for Tea Improvement, Tea Research Institute, Chinese Academy of Agricultural Sciences (TRICAAS), 9 Meiling South Road, Hangzhou 310008, China

## Abstract

Tea (*Camellia sinensis*) is widely cultivated throughout the world for its unique flavor and health benefits. Galloylated catechins in tea plants serve as important secondary metabolites that play a pivotal role in tea taste determination and pharmacological effects. However, the genetic basis of galloylated catechins traits remains elusive. We identified a stable and major-effect quantitative trait locus (QTL) associated with galloylated catechins index (GCI), designated *qGCI6.2*. Within the QTL’s confidence interval, two shikimate dehydrogenases (*CsSDH4*, *CsSDH3*) were identified. These enzymes catalyze gallic acid (GA) production from 3-dehydroquinate dehydratase, thereby contributing to galloylated catechins accumulation. Quantitative real-time PCR (RT-qPCR) analysis revealed that *CsSDH4* and *CsSDH3* expression levels and GA and galloylated catechins contents were positively correlated. Furthermore, overexpressing *CsSDH4* and *CsSDH3* in transgenic tomato plants markedly increased GA and galloylated catechin contents. RNA-seq analysis of transgenic tomato indicated that *CsSDH4* and *CsSDH3* primarily regulate genes related to shikimic acid and flavonoid pathways, and jointly promote galloylated catechins synthesis. Our findings have further elucidated the galloylated catechins synthesis pathway and provided a theoretical basis for cultivation of tea cultivars with high galloylated catechin contents.

## Introduction

**Figure 1:**
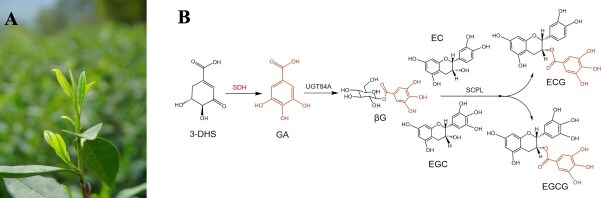
Biosynthetic pathways of galloylated catechins in tea plants. (A) Phenotypes of the elite green tea variety ‘LJ43’. (B) Biosynthetic pathways of galloylated catechins. 3-DHS; 3-dehydroshikimate; GA, gallic acid; 1-O-β-glucogallin, βG; *SDH*, shikimate dehydrogenases (the gene found in this study); *SCPL*, serine carboxypeptidase-like acyltransferases.

Tea is globally one of the most popular nonalcoholic beverages. Tea popularity is mainly due to its distinct flavor properties and health benefits, as tea leaves contain a variety of bioactive components, such as amino acids and catechins (flavan-3-ols) [1]. Tea catechins are the major flavonoids, accounting for 12%–24% of dry weight in fresh leaves (Fig. 1a). There are seven main catechins in tea: (+)-catechins (C); (−)-epicatechin (EC); (+)-gallocatechin (GC); (−)-epigallocatechin (EGC); (−)-gallocatechin gallate (GCG); (−)-epicatechin-3-gallate (ECG); and (−)-epigallocatechin-3-gallate (EGCG), according to their chemical structural formulae. Among them, EGCG, ECG, EGC, and EC are the most abundant catechins, accounting for >90% of total catechins amounts [2]. EGCG and ECG are collectively referred to as galloylated catechins, while EGC and EC are collectively referred to as nongalloylated catechins. Galloylated catechins are more important than nongalloylated catechins for two main reasons: (1) the contribution of galloylated catechins to tea flavor is greater than nongalloylated catechins, as galloylated catechins give green tea a bitter and astringent flavor [3]; and more importantly, (2) galloylated catechins have a variety of benefits to human health, such as neuroprotective [4, 5], antioxidant [6, 7], antiinflammatory [8], anticancer ,[9], and anti-COVID-19 properties [10]. Therefore, galloylated/nongalloylated catechins (as galloylated catechins indices, GCIs) are an important agronomic trait [11].

The regulatory pathway for galloylated catechins biosynthesis in tea plants has been elucidated (Fig. 1b). A comprehensive analysis of the transcriptome and metabolite correlations revealed a strong association between tissue-specific expression patterns of 14 *CsSCPL* genes and EGCG and ECG formation [12]. Moreover, coexpression of *CsSCPL4* and *CsSCPL5* in the presence of 1-O-β-glucogallin (βG), catalyzes ECG and EGCG synthesis from nongalloylated catechins (EC, EGC) [13]. This underscores that galloylated catechins synthesis requires two crucial precursors: nongalloylated catechins and βG. Notably, the nongalloylated catechins biosynthetic pathway has been increasingly elucidated in tea plants [14–17]. Meanwhile, *CsUGT84A22* ability to directly catalyze βG production from gallic acid (GA) has been reported [18, 19]. Additionally, three *VvgGTs* from grapes and *VvUGT84A13* in pedunculate oak are catalytically active in βG formation, respectively [20, 21]. Moreover, GA, the precursor substance for βG synthesis, has been reported in other crops. GA can be synthesized from 3-dehydroshikimate (3-DHS) catalyzed by shikimate dehydrogenases (*SDH*) enzyme activity with NADP^+^ as a cofactor [22]. In walnut, functional characteristics of *JrSDH*, which was catalytically active on 3-DHS to produce GA, have been reported [23]. However, despite the economic importance of tea plants, which are rich in galloylated catechins, GA biosynthesis in tea plants is still poorly understood. There is an urgent need to investigate the functional properties of *SDH* and its role in galloylated catechins regulation.

In this study, a quantitative trait locus (QTL) analysis was conducted on the GCI trait in 327 F_1_ individuals from a LJ43 × BHZ population, then a major and stable QTL (*qGCI6.2*), which was observed over 3 years, was identified (2020 phenotypic variation explained (PVE) = 8.7%, 2021 PVE = 14.73%, and 2022 PVE = 15.80%). Furthermore, within the candidate QTL region, *CsSDH3* and *CsSDH4* were identified as key candidate genes. In 16 F_1_ individuals, *CsSDH4* and *CsSDH3* expression levels were highly correlated with GA content, and also strongly correlated with GCI. Additionally, overexpressing both *CsSDH* genes in tomato led to a significant elevation in GA and flavonoid contents. In tea, we not only identified *CsSDH4* and *CsSDH3* gene function but also shed light on the galloylated catechin synthesis pathway. Our work lays a foundation for further development of tea cultivars rich in galloylated catechins.

## Results

### QTL mapping results associated with GCI

**Figure 2:**
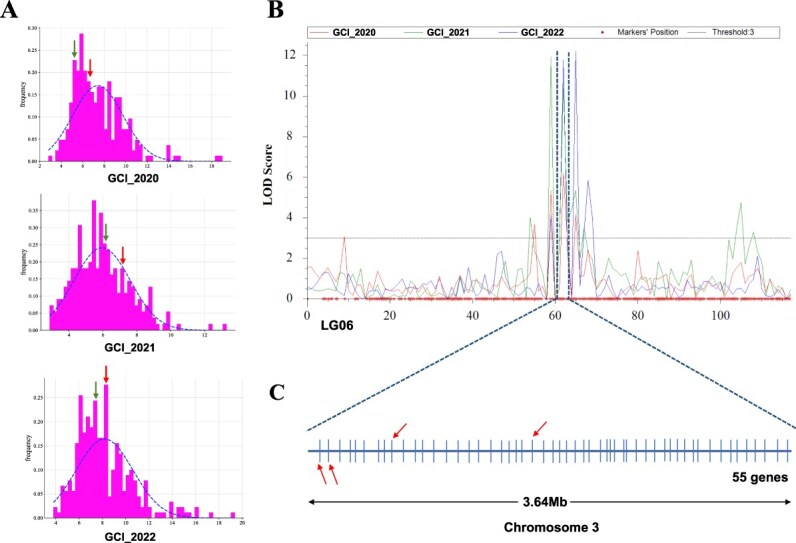
Phenotypic observation the GCI trait and fine-mapping of the major-effect QTL. (A) Phenotype of the GCI traits of 327 individuals of LJ43 × BHZ from 2020 to 2022. The green arrow represents female parent LJ43, the red arrow represents male parent BHZ. (B) Inclusive composite interval mapping of the major-effect QTL (*qGCI6.2*) on linkage group 06. Different color curves represent LOD values for GCI trait in different years, GCI_2020 (red), GCI_2021 (green), GCI_2022 (blue). (C) The 55 genes on chromosome 3 of the tea reference genome. Four key candidate genes for *CsSDH* are shown as red arrows.

To understand the genetic basis of tea catechins, we constructed a 327-progeny population by crossing LJ43 × BHZ in 2011 [24]. We collected one bud and two leaves from individuals of the population and their parents in spring 2020, 2021, and 2022. Catechins compounds content was then measured by high-performance liquid chromatography (HPLC) (Table S1). Meanwhile, the GCI trait (ratio of galloylated catechins to nongalloylated catechins) was also calculated (Fig. 2a, Table S2). GCI ranged from 2.81 to 18.9 (2020), 2.87 to 13.28 (2021), and 3.80 to 19.35 (2022), while coefficients of variation were 27.80%–31.70%, respectively. These results indicate that GCI showed extensive segregation in the progeny population.

QTL mapping of GCI traits in the whole genome was performed using GACD 1.2 software. A major and stable QTL (*qGCI6.2*) was identified at 61.83–62.45 cM of LG06 (Fig. 2b), with a logarithm of odds (LOD) score of 6.20–11.76. PVE ranged from 7.30% to 15.80% in the three consecutive years (Table S3).

To discover the key candidate genes, probe sequences (~71 bp) of markers flanking *qGCI6.2* were blasted to the ‘Shuchazao’ reference whole tea genome [25]. In our previous work, we reported that the genetic linkage map was highly consistent with the tea genome [26]. Thus, *qGCI6.2* location at 78.01–81.66 Mb on chromosome 3 of the ‘SCZ’ tea genome was obtained (Fig. 2c), and 55 genes in the interval were identified (Table S4). By reviewing the literature and gene function annotations, shikimate dehydrogenases were identified as important enzymes in the galloylated catechin synthesis pathway [27, 28]. Therefore, four shikimate dehydrogenases, namely CSS0042855 (*CsSDH4*), CSS0006477 (*CsSDH3*), CSS0027964 (*CsSDH2*), and CSS0030581 (*CsSDH1*) were identified as the key candidate genes for galloylated catechins.

### Cloning of *CsSDH* candidate genes in tea plants

**Figure 3:**
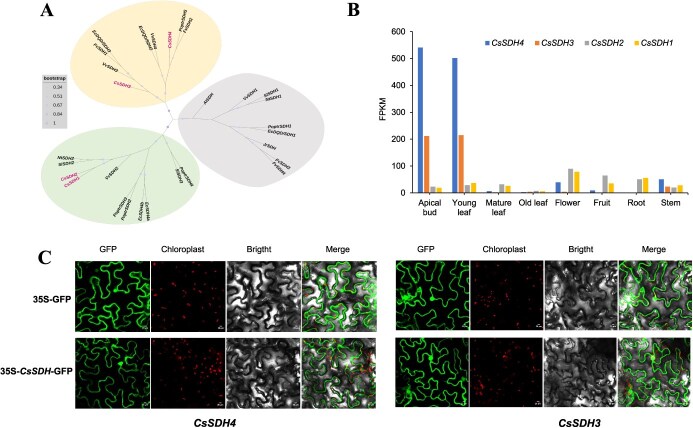
Phylogenetic, transcription levels, and subcelluar localization of *CsSDHs*. (A) Phylogenetic analysis of plant DQD/SDH protein sequences in tomato and other species by the neighbor-joining method. At, *Arabidopsis thaliana*. Cs, *C. sinensis*. Sl, *Solanum lycopersicum*. Nt, *Nicotiana tabacum*. Fv, *Fragaria vesca*. Vv, *Vitis vinifera*. Poptr, *Populus trichocarpa*. Ec, *Eucalyptus camaldulensis*. Bootstrap values are shown next to the nodes by dots. (B) *CsSDH1–4* transcription levels in different tissues by RNA-seq analysis. Data from TPIA (http://tpia.teaplants.cn/) database. (C) Subcelluar localization of *CsSDH4* and *CsSDH3* in tobacco leaf epidermal cells. The 35S-GFP vector was used as the control, GFP signal is shown as green fluorescent protein. The scale bar in the graph is 20 μM.

To explore the function of four *CsSDH* genes in tea plants, SDH multiple sequence alignment analysis in tea plants was performed with other species. The CsSDHs protein sequence from tea genome ‘Shuchazao’ and Arabidopsis, tobacco, eucalyptus, tomato, grape, strawberry, and walnut protein sequences were downloaded from NCBI. The four CsSDH protein sequences were divided into two main groups, with VvSDH4, EcDQD/SDH2 and VvSDH3, EcDQD/SDH3 closer to CsSDH4, CsSDH3 proteins, respectively (Fig. 3a)*.* The functions of these genes in GA and flavonoid synthesis have been reported [23, 27]. Interestingly, when we used AtSDHs to search for homologous genes in tea plants, only five CsSDH proteins were found. With the exception of CSS0015872, through QTL mapping, we precisely identified the remaining four *CsSDH* genes that play an important role in GA synthesis in tea plants.

We further analyzed the gene expression patterns of the four *CsSDH* genes in different tea plant tissues (apical bud, young leaf, and mature leaf) (Fig. 3b). *CsSDH4* and *CsSDH3* exhibited a comparable gene expression pattern, which was significantly higher in apical buds and young leaves compared to other tissues. This pattern is consistent with the accumulation of GA and galloylated catechin content, which is mainly concentrated in young shoot tissues [12, 29]. This alignment suggests a significant role of these two genes in galloylated catechin synthesis, prompting us to select them for further investigation.

### Subcellular localization

To gain insight into *CsSDH4* and *CsSDH3* subcellular localization, we cloned the coding sequences (CDS) of these two genes from LJ43. We then fused the *CsSDH4* and *CsSDH3* genes to the 35S-GFP vector, creating constructs designated as 35S:*CsSDH4*-GFP and 35S:*CsSDH3*-GFP. These constructs were subsequently transformed into *Agrobacterium tumefaciens* strain GV3101 and used for infiltration into tobacco leaves. Both CsSDH4 and CsSDH3 proteins were localized in the cytoplasm and nucleus (Fig. 3c).

### CsSDH4 and CsSDH3 have specific activity on 3-DHS-generated GA

To validate that GA was directly synthesized from 3-DHS and catalyzed by CsSDH4 and CsSDH3, we fused these enzymes to a maltose-binding protein and expressed the fusion constructs in *Escherichia coli* Novablue (DE3) competent cells. The purification results of the recombinant protein are shown in Fig. S1a**.** To assess CsSDH4 and CsSDH3 enzymatic activity, crude enzymatic reactions were conducted using coenzyme NADP^+^ and 3-DHS as substrates. GA produced in these reactions was quantified via HPLC. The results conclusively demonstrated that GA was directly generated under the enzymatic catalysis of the CsSDH4 and CsSDH3 recombinant proteins (Fig. S1b).

**Figure 4:**
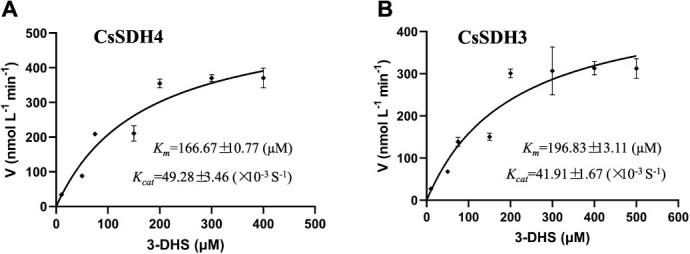
Kinetic analysis of CsSDH4 (A) and CsSDH3 (B) enzymes using 3-DHS as substrate.

To further investigate CsSDH4 and CsSDH3 recombinant protein enzymatic properties, we first investigated their optimal reaction conditions. For CsSDH3 they were 40°C and pH 8.0 and for CsSDH4, 35°C and pH 8.0 (Fig. S1c, d**).** The Michaelis–Menten equation was used to fit the curves and calculate the enzyme kinetic parameters (Fig. 4). With 3-DHS as the substrate, CsSDH4 and CsSDH3 *Km* values were 166.67 and 196.83 μM, and *Kcat/Km* values were 295.63 and 213.34 M^−1^S^−1^, respectively. CsSDH4 showed higher substrate affinity and catalytic efficiency for 3-DHS as substrate compared to CsSDH3.

### GA and galloylated catechins content are closely related in *CsSDH4* and *CsSDH3*

**Figure 5:**
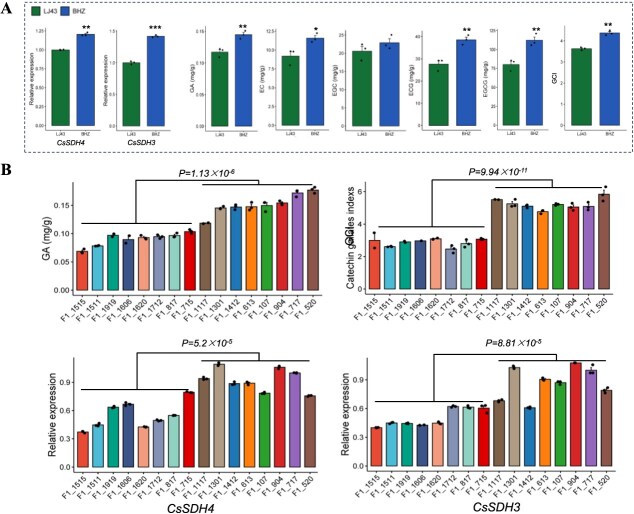
GA and catechins content associated with *CsSDH4* and *CsSDH3* expression levels. Values represent three biological replicates (means ± SD). (A) GA, catechins content and level expression of *CsSDH4* and *CsSDH3* in parents LJ43 and BHZ. (B) Correlations between GA content, GCI, and *CsSDH4* and *CsSDH3* expression levels in 16 individuals of the F_1_ population.

To understand *CsSDH4* and *CsSDH3* as well as their association with GA and catechins content, we also measured catechins fractions content. Furthermore, we measured *CsSDH4* and *CsSDH3* transcript levels in one bud and two leaves of the 16 F_1_ individuals of the LJ43 × BHZ population and their parents. The relative expressions of *CsSDH4* and *CsSDH3* in BHZ were significantly higher than in LJ43 (Fig. 5a). Meanwhile, GA, EC, EGC, ECG, and EGCG contents and GCI traits in BHZ were also significantly higher than in LJ43. Additionally, in the 16 F_1_ generations with extreme GA content phenotypes (Table S5), the same GCI phenotypes as well as *CsSDH4* and *CsSDH3* expression levels were also significantly different (Fig. 5b). *CsSDH4* and *CsSDH3* expression levels were significantly and positively correlated with GA content (*R* = 0.80, *P* = 0.00019; *R* = 0.85, *P* = 2.8 × 10^−5^) and GCI (*R* = 0.81, *P* = 0.00015; *R* = 0.85, *P* = 0.00064), respectively (Fig. S2). These results suggest that *CsSDH4* and *CsSDH3* are essential genes for GA synthesis regulation, and GA may also be involved in galloylated catechins synthesis.

### Functional characterization of *CsSDH4*, *CsSDH3* in transgenic plants

**Figure 6:**
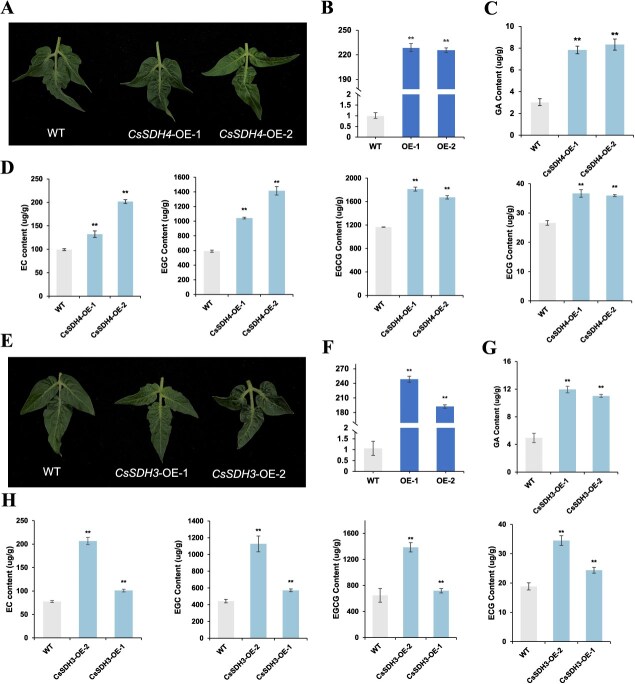
Catechin contents and *CsSDH4* and *CsSDH3* expression levels in tomato lines. Values are means ± SD. *P < 0.05, **P < 0.01 (Student’s *t*-test). (A) leaf phenotype of overexpressed *CsSDH4* and WT tomato lines, (B) qRT-PCR results of *CsSDH4* in WT and CsSDH4 overexpression lines, (C) GA content in WT and *CsSDH4* overexpression lines, (D) flavonoid content in WT and *CsSDH4* overexpression lines, (E) leaf phenotype of overexpressed *CsSDH3* and WT tomato lines, (F) qRT-PCR results of *CsSDH3* in WT and *CsSDH3* overexpression lines, (G) GA content in WT and *CsSDH3* overexpression lines, (H) flavonoid content in WT and *CsSDH3* overexpression lines.

We determined GA and EC, EGC, ECG, and EGCG contents in ‘Micro-Tom’ tomato plants (Fig. S3). The presence of tea catechins in tomato plants has been reported in previous studies [30]. In the absence of a successfully established tea plant genetic transformation system, transgenic tomato appears to be an ideal model crop to validate secondary metabolism regulatory genes instead of ontogenetic transformation of tea plants. Therefore, to validate this suspicion, CaMV35S promoter was used to heterologously overexpress *CsSDH3* and *CsSDH4* in ‘Micro-Tom’ tomato plants to investigate their role in the catechin synthesis pathway (Fig. 6a, e). In the transgenic lines, *CsSDH4* and *CsSDH3* gene expression levels increased ~180- to 250-fold compared to the wild-type (WT) plants (Fig. 6b, f). As expected, the transgenic lines (*CsSDH4-OE1*, *CsSDH4-OE2*, *CsSDH3-OE1*, and *CsSDH3-OE2*) showed a significant increase in GA content (Fig. 6c, g). Simultaneously, EC, EGC, ECG, and EGCG contents all increased in leaves of the transgenic lines (Fig. 6d, h). These findings indicate that *CsSDH4* and *CsSDH3* positively regulate GA content, a compound that subsequently plays a crucial role in catechin synthesis.

### Transcriptomic analysis of *CsSDH4* and *CsSDH3* transgenic tomato

**Figure 7:**
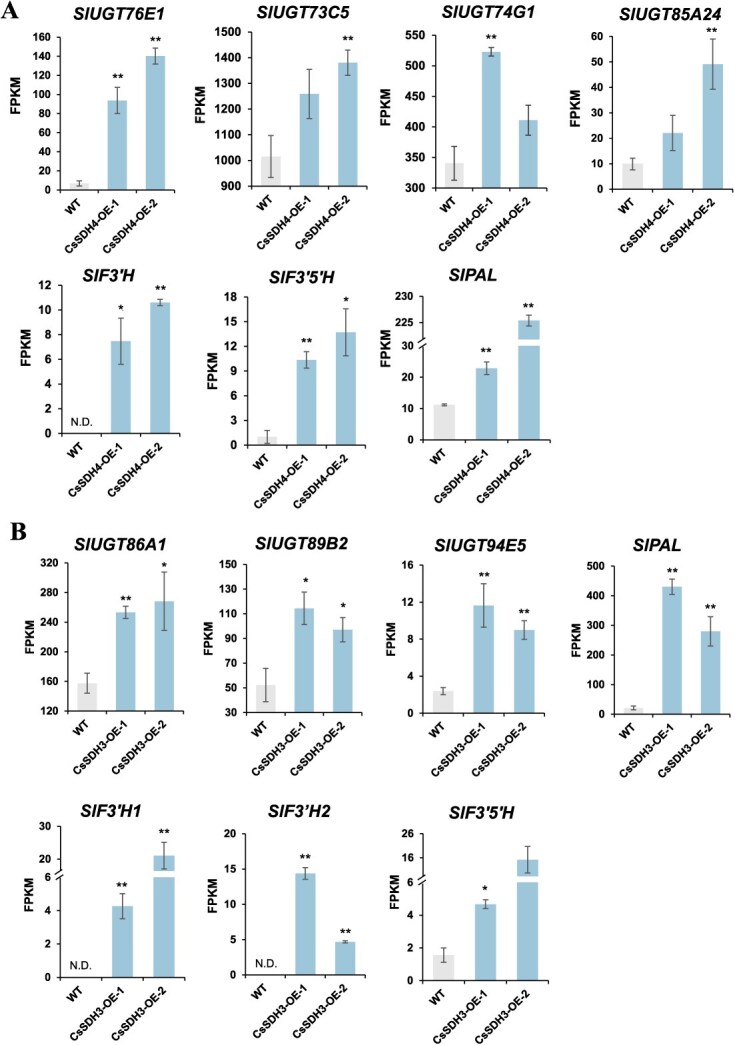
Transcription levels of DEGs associated with flavonoid biosynthesis in WT and *CsSDH4* and *CsSDH3* overexpression lines. Values are means ± SD. *P < 0.05, **P < 0.01 (Student’s *t*-test). The expression level of flavonoid biosynthetic genes in (A) WT and *CsSDH4* overexpression lines, and (B) in WT and *CsSDH3* overexpression lines.

The genes encoding UDP-glucosyltransferase (*UGT*), flavanone-3′-hydroxylase (*F3’H*), flavanone-3′5’-hydroxylase (*F3’5’H*), and Phenylalanine ammonia-lyase (*PAL*) play pivotal roles in flavonoid biosynthesis. To uncover the underlying *CsSDH4* and *CsSDH3* molecular mechanisms, we used RNA-seq to analyze *CsSDH4* and *CsSDH3* transgenic plants and WT tomato leaves. A total of 1196 (*CsSDH4-OE* vs WT) and 2283 (*CsSDH3-OE* vs WT) differentially expressed genes (DEGs) were identified (Fig. S4). Among these upregulated genes (Table S6, Table S7), *SlUGTs*, *SlF3’H*, *SlF3’5’,H*, and *SlPAL* transcript levels were all significantly induced in *CsSDH4-OE* and *CsSDH3-OE* compared to WT (Fig. 7). These results demonstrate that *CsSDH4* and *CsSDH3* overexpression could significantly influence transcriptional activity of genes associated with flavonoid biosynthesis in tomatoes.

**Figure 8:**
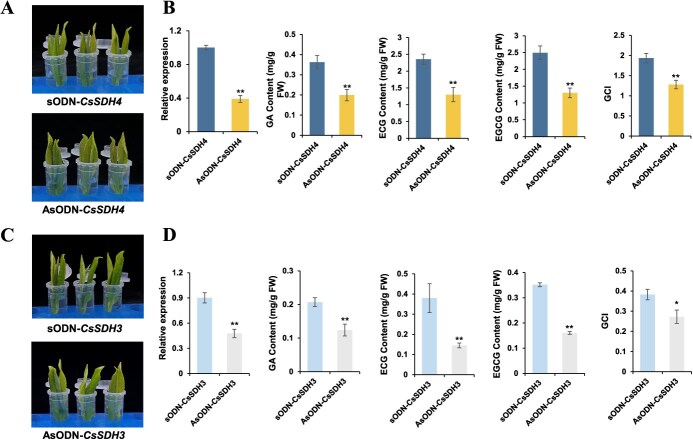
Antisense oligonucleotide silencing analysis of *CsSDH4* and *CsSDH3* in tea plants. Values represent three biological replicate means ± SD. *P < 0.05, **P < 0.01 (Student’s *t*-test). (A) *CsSDH4* antisense oligonucleotide analysis, (B) *CsSDH4* expression level and catechins content in RNAi treatment of *CsSDH4,* (C) *CsSDH4* antisense oligonucleotide analysis, (D) *CsSDH3* expression level and catechins content in RNAi treatment of *CsSDH3*.

To further validate *CsSDH4* and *CsSDH3* functions in tea plants, given the absence of a stable genetic transformation system, we used RNAi technology to knock down *CsSDH4* and *CsSDH3* (Fig. 8a, c). There was a significant downregulation in both *CsSDH4* and *CsSDH3* expression levels in antisense oligonucleotide (AsODN)-treated leaves compared to the sense ODN treatment (Fig. 8b, d). Consistent with gene expression levels, GA, galloylated catechins, and GCI contents were also significantly reduced in the AsODN-treated samples compared to the sense ODN treatment (Fig. 8b, d).

## Discussion

### Advances in QTL mapping associated with GCI traits

Galloylated catechins, serving as important functional active compounds in tea, not only exert profound impacts on tea flavor and quality but also provide numerous human health benefits. Therefore, they have a crucial influence on tea quality and economic value [31]. Recently, the galloylated catechins synthesis and hydrolysis pathways have been reported, such as *CsUGT84A22*, and *CsSPL4* and *CsSPL5* coexpression have been shown to contribute significantly to ECG and EGCG synthesis [13, 18]. However, there are few reports on galloylated catechins with QTL mapping using classical genetic methods. Previously, we conducted QTL studies on flavonoids and identified 27 flavonoid-related QTLs [32]. However, due to insufficient molecular markers in the genetic map, fine mapping of candidate genes linked to flavonoids remains a challenge. Fortunately, we recently addressed this issue through the development of a 200 K tea SNP array, resulting in a high-density genetic map with an average interval of 0.39 cM [26]. Furthermore, this genetic map was successfully used to identify the *CsAGL9* gene, which regulates the number of seed set in tea plants [33]. Therefore, it is an efficient platform for future genetic research in tea plants [26].

### Functional analysis and characterization of the *CsSDHs* genes

Based on the constructed high-density genetic map, and after three consecutive years of experimental analysis, we successfully identified a major and stable QTL, *qGCI6.2*, which is intricately related to GCI traits. The QTL interval physical distance in the reference tea genome [25] was 3.64 Mb and contained 55 genes. Further investigation of gene function and tissue-specific expression patterns narrowed the search to two pivotal candidate genes: *CsSDH4* and *CsSDH3*.

The enzyme dehydroquinate dehydratase/shikimate dehydrogenase (DQD/SDH) plays a crucial role in GA biosynthesis, an intermediate metabolite that contributes significantly to the formation of polyphenolic compounds in plants [22]. *In vitro* assays of the catalytic function of SDHs in GA formation have been reported in many species, such as grape (*VvSDH3*, *VvSDH4*) [27] and eucalyptus (*EcDQD/SDH3*, *EcDQD/SDH2*) [34]. Intriguingly, the phylogenetic analysis of DQD/SDH proteins revealed a close relationship between the CsSDH3, CsSDH4 proteins and their counterparts in grape (VvSDH3, VvSDH4) and eucalyptus (EcDQD/SDH3, EcDQD/SDH2). This finding further supports their functional similarity, and also confirmed CsSDH4 and CsSDH3 enzyme activity in GA production. Furthermore, there is a synchronized expression pattern between *VvSDH3* and *VvSDH4* with GA biosynthesis in grape berries [27]. Our results are similar to previous reports; in LJ43 and BHZ and their F_1_ progeny populations, *CsSDH4* and *CsSDH3* transcript levels were significantly and positively correlated with GA content and GCI traits, respectively.

In persimmon, fruits derived from nonastringent-type mutants exhibited significantly lower levels of GA, ECG, and EGCG compared to their astringent counterparts [35]. Moreover, the *DkSDH* gene expression profile was significantly reduced in these nonastringent fruits [36]. Analogously, a nonastringent Chinese cultivar, ‘LuoTianTianShi’, also displayed *DkSDH* downregulation [37]. Nevertheless, the precise roles of CsSDH4 and CsSDH3 in modulating galloylated catechins synthesis in tea plants have yet to be conclusively demonstrated. In this study, *CsSDH4* and *CsSDH3* overexpression in transgenic tomato increased GA and galloylated catechins contents. Conversely, transient silencing of these two genes in tea plants significantly decreased GA and galloylated catechins contents. These results demonstrate a pivotal role of *CsSDH4* and *CsSDH3* in positively regulating GA and galloylated catechins contents in tea plants. Based on the results of enzyme activities and gene expression levels, the role of *CsSDH4* is more important than *CsSDH3* in tea plants. This result was similar to the results of *VvSDH4* and *VvSDH3* studies in grapes [27].

### Biosynthetic pathways of galloylated catechins

Flavan-3-ol gallates, whose major components include EGCG and ECG, are abundant in many crops such as persimmons [35], grapes [38], and tea plants [39]. These compounds impart a nutritious bitter and astringent flavor to fruits and beverages [3]. Overexpression of *VvSDH3* in grape hairy roots resulted in an increase in GA, βG, and flavan-3-ol gallate. Subsequently, the GA underwent glycosylation and was converted to flavan-3-ol, ultimately leading to GA accumulation in the form of flavan-3-ol gallate [27]. Therefore, the main reason why *CsSDHs* affect galloylated catechins contents is primarily due to their ability to synthesize GA, which subsequently serves as the source of gallic groups for galloylated catechins. Recently, a UDP-glucosyltransferase (*UGT*) enzyme *CsUGT84A22,* which catalyzes the glycosylation of gallic acid to produce βG, was identified in tea plants [18]. In our study, compared to WT, several *SlUGTs* were significantly upregulated in transgenic tomatoes, such as *SlUGT76E1*, *SlUGT73C5*, *SlUGT74G1*, and *SlUGT85A24*. Therefore, we hypothesized that these *SlUGTs* may be involved in galloylated catechin biosynthesis in tomato by catalyzing the generation of βG from GA. Meanwhile, the significant upregulation of flavonoid biosynthesis-related gene expression in *CsSDH4* and *CsSDH3* overexpression tomatoes increased flavonoid (EC, EGC, ECG, and EGCG) production, which was consistent with flavonoids accumulated in *SlSDH2* overexpression tomato fruits [40]. Interestingly, identified through WGCNA analysis, the top 200 genes most closely associated with *CsSDH4* and *CsSDH3* (Table S8) included *CsUGT73B5* (CSS0035776), *CsF3’5’H* (CSS0014132, CSS0022212), flavonol synthase (*CsFS*, CSS0033075, CSS0046529), and chalcone synthase (*CsCHS*, CSS0004474, CSS0007714, CSS0030597) from tea plants. These structural genes play a crucial role in the flavonoid synthesis pathway [12]. These results indicate that *CsSDH4* and *CsSDH3* primarily affect galloylated catechins accumulation by regulating the shikimate and flavonoid synthesis pathways in tea plants.

## Conclusions

We successfully cloned the first QTL associated with the tea plant GCI trait, *qGCI6.2*, and further elucidated the pivotal roles of *CsSDH4*, *CsSDH3* in GA and galloylated catechin biosynthesis. Furthermore, we discovered that, in addition to regulating GA biosynthesis, *CsSDH4* and *CsSDH3* also induced flavonoid synthesis, ultimately increasing galloylated catechin contents. These results clarify the galloylated catechin synthesis pathway in tea plants, while laying a foundation for further development of tea cultivars rich in galloylated catechins.

## Materials and methods

### Plant materials

The segregating population consisted of 327 F_1_ individuals produced by crossing the elite green tea cultivar ‘Longjing 43’ (LJ43) and ‘Baihaozao’ (BHZ), with LJ43 as the female parent, and BHZ as the male parent. All individuals were planted at the germplasm resources facility of the Tea Research Institute, Chinese Academy of Agricultural Sciences (TRICAAS), Shengzhou, China. For further details see Wang *et al.*, [33]. The same fertilizer and water management practices were applied to all individual plants.

### Phenotyping of GCI traits

In spring 2021, 2022, and 2023, one bud and two leaves were collected from the parents and their progeny of 327 individuals. The samples were first collected and processed in a hot air dryer at 120°C for 10 min. They were then baked at 80°C until they reached a constant weight. Then, they were ground into powder and stored in a refrigerator at −20°C for future use.

The contents of seven catechin compounds (GC, EGC, C, EC, EGCG, GCG, and ECG) in a genetic population were determined using HPLC. Detailed analysis conditions and gradient elution procedures (with minor modifications) are described in Wei et al., [41]. Briefly, a weighed 0.10-g sample was placed in a 10-ml centrifuge tube, and 5 ml of 70% methanol solution was added. The mixture was allowed to extract for 20 min in a water bath maintained at 70°C (the tube was shaken every 5 min), then cooled to room temperature. Then, the solution was centrifuged at 3500 rpm for 10 min. One milliliter of the supernatant was pipetted into a separate 5-ml centrifuge tube and 1 ml of stabilizing solution was added. The contents were thoroughly mixed, then filtered through a 0.45-μm organic microporous membrane. To evaluate the galloylated catechins, we calculated an index defined as the ratio of (EGCG + ECG) to (EC + EGC). Detailed results of this analysis are listed in Tables S1 and S2.

### QTL mapping and candidate gene search

The high-density genetic mapping employed in this study has been previously reported [26]. The map contained 5325 markers, evenly covering 15 linkage groups (LGs), with an average marker distance of 0.39 cM. QTL mapping associated with GCI was performed for 327 F_1_ individuals using GACD1.2 software. An LOD threshold of 3.0 was used to identify the QTL associated with the GCI trait [42], and QTLs with the same two flanking markers were defined as the same QTL. To define information about candidate genes within the QTL interval, the sequences of two flanking SNP markers were mapped to the tea reference genome ‘Shuchazao’ by TeaGVD (http://www.teaplant.top/teagvd). Expression levels of candidate genes in eight different tea plant tissues were determined from TPIA (Table S4) [25].

### Phylogenetic analysis of *CsSDHs*

To further analyze the protein sequences of tea plant *CsSDHs*, the protein sequences of grape, *Arabidopsis*, and other crops were downloaded from NCBI for phylogenetic tree analysis construction. The accession numbers are *AtSDH* (AAF08579), *JrSDH* (AAW65140), *FvSDH1* (XP_004302480), *FvSDH2* (XP_004302479), *FvSDH3* (XP_004289250), *FvSDH4* (XP_004288087), *NtSDH1* (AAS90325), *NtSDH2* (AAS90324), *PoptrSDH1* (Potri.010G019000), *PoptrSDH2* (Potri.013G029900), *PoptrSDH3* (Potri.005G043400), *PoptrSDH4* (Potri.014G135500), *PoptrSDH5* (Potri.013G029800), *VvSDH1* (KU163040), *VvSDH2* (KU163041), *VvSDH3* (KU163042), *VvSDH4* (KU163043), *EgSDH1* (Eucgr.H01214.1), *EgSDH2* (Eucgr.H04428.1), *EgSDH3* (Eucgr.H04427.1), *EgSDH4* (Eucgr.B01770.2), and *EgSDH5* (Eucgr.J00263.6). The neighbor-joining method was used to construct a phylogenetic tree of 1000 bootstrap replicates using MEGA-7 software. Phylogenetic trees were visualized and edited using the online web ITOL (https://itol.embl.de).

### Subcellular location

Full-length CDS of *CsSDH4* and *CsSDH3* were cloned from the LJ43 for subcellular localization analysis. The plasmid 35S-*CsSDH4*-GFP, 35S-*CsSDH3*-GFP, and 35S-GFP (control) were transformed into *A. tumefaciens* (GV3101), respectively. *Agrobacterium* cells were cultured in solid media containing kanamycin and rifampicin. Single colonies were selected and cultured in liquid media. The bacteria were suspended in 10 mM Mgcl_2_ to OD600 = 0.6. *Agrobacterium* cells were injected into the back of tobacco leaves, cultured for 2 days in a low-light environment, then observed and photographed under a confocal laser microscope (Nikon C2-ER, Nikon, Japan).

### Analysis of CsSDH4 and CsSDH3 enzyme activity

CsSDH4 and CsSDH3 coding sequences were cloned into the pET-32a expression vector and the recombinant vector was transformed into *E. coli* BL21(DE3) to expression recombinant protein. The cells were cultivated in lysogeny broth (LB) medium until cell density reached OD_600_ = 0.6. Subsequently, 1 mM IPTG was added to the medium and maintained at 24°C for 24 h to induce protein expression. After induction, the precipitate was collected; resuspended in buffer consisting of 200 mM NaCl, 20 mM Tris, 1 mM EDTA, and 1 mM DTT; and pH was adjusted to 7.5. This was followed by centrifugation at 6500 rpm for 15 min to separate the supernatant. Subsequently, we performed a crude enzyme activity analysis using reference literature [28], and reaction products in the enzymatic assays were quantified by HPLC.

Purification of target proteins was achieved using a His-Tagged Protein Purification Kit (Cwbio, Jiangsu, China), and recombinant protein concentrations were determined using a Bradford protein quantification kit (Aidlab, Beijing, China). Optimal reaction conditions for recombinant proteins were determined after evaluating the effects of different reaction temperatures and pH. For determination of the optimal pH of the proteins, pH gradients (6.5, 7, 7.5, 8, and 8.5) for the substrate buffer were based on a standard reaction program. Optimum temperature for the enzyme was based on a standard reaction program with a gradient of reaction temperatures for the enzyme (25°C, 30°C, 35°C, 40°C, 45°C, and 50°C). Briefly, the reaction was conducted at 40°C for 30 min in a buffer consisting of 100 mM Tris-HCl (pH 8.0), with a substrate of 1mM of 3-DHS, 1 mM NADPH, and included 10 µg of purified recombinant protein CsSDH4/3.. The reaction was then terminated by adding 100 μl of methanol, and reaction products in the enzymatic assays were quantified by liquid chromatograph-mass spectrometer/mass spectrometer (LC–MS/MS).

*In vitro*, determination of kinetic parameters of CsSDH4/3 under optimal conditions. The 100-μl reaction mixture contained 100 Mm Tris–HCl buffer (pH 8.0), 1 mM NADPH, 0-500 mM of 3-DHS as the substrate, and 10 μg purified recombinant protein CsSDH4/3. The reaction was conducted at 40°C for 30 min. The reaction was then terminated by adding 100 μl of methanol. Three biological replicates were used per reaction. Enzyme kinetic parameters (*Km*, *kcat*, and *kcat/Km*) were obtained by fitting Michaelis–Menten equation curves (for nonlinear regression analysis) using GraphPad Prism 8.

### Construction of transgenic tomato-overexpressing *CsSDH4* and *CsSDH3*

To conduct tomato genetic transformation, we employed *Agrobacterium*-mediated transformation methods [43]. Specifically, the CDS sequences of *CsSDH4* and *CsSDH3* were precisely inserted into the pBWA(V)HS vector by homologous recombination, resulting in the construction of 35S-*CsSDH4* and 35S-*CsSDH3* vectors, respectively. The successfully constructed vectors were then efficiently transferred into *A. tumefaciens* competent cells (strain GV3101). The positive *Agrobacterium* colonies were selectively cultured on LB solid medium containing kanamycin (50 μg/ml). Subsequently, ‘Micro-Tom’ tomato (WT) explants were infiltrated with the *A. tumefaciens* liquid culture. Finally, hygromycin screening and RT-qPCR were used to identify and select positive transgenic lines. The tomato plants were transferred to a plant culture chamber and incubated in a 14-h light/10-h dark photoperiod and 25°C/18°C temperature cycle with regular watering. Sample collection and observation were carried out after 110 days.

### RNA-seq analysis

*CsSDH4*, *CsSDH3* overexpression lines and WT leaves were used for transcriptome sequencing analysis. Total RNA was extracted using a plant RNA extraction kit (Aidlab, Beijing, China), 1% agrose gel electrophoresis was used to check RNA quality, and NanoDrop 2000 (Thermo Scientific, Beijing, China) was used to determine RNA concentration. The high-quality RNA was selected to be sent to Beijing Novogene Co., Ltd (Beijing, China) for sequencing in an Illumina NovaSeq platform. To ensure data analysis quality and reliability, the raw data were filtered. The main purpose was to remove the reads with adapters, unidentifiable bases, and those of low quality to obtain clean data, which were compared with the tomato reference genome using HISAT2 v2.0.5 software [44]. FPKM values were calculated for each gene based on gene length and the number of reads mapped to it. Subsequently, DEGs were analyzed between the two comparative combinations using DESeq2 software (1.20.0) to identify DEGs at a significance threshold of *P* < 0.05. Furthermore, the raw sequencing data we obtained were deposited into the NCBI Sequence Read Archive under the BioProject accession no. PRJNA1128726.

### Gallic acid and flavonoid analysis in tomatoes

LC–MS/MS (Waters, USA) was used for gallic acid and catechin determination. The chromatographic column was Waters ACQUITY UPLC BE-H-C18 (2.1 × 100 mm, 1.8 μm). The mobile phases were 0.1% formic acid in water (C) and 100% acetonitrile (D). Linear gradient elution: 0–5 min, 98% C, 2% D; 5–10 min, 98%–90% C, 2%–10% D; 10–15 min, 90%–80% C, 10%–20% D; 15–17 min, 80%–75% C, 20%–25% D; 17–17.10 min, 75%–0% C, 25%–100% D; 17.10–18.60 min, 0% C, 100% D; 18.6–18.7 min, 0%–98% C, 100%–2% D; 18.7–20 min, 98% C, 2.0% D; flow rate of 0.28 ml/min, column temperature 35°C, injection volume of 5 μl, running for 20 min.

Mass spectrometry conditions: ESI (electrospray ionization), positive ion mode scanning, 3.5 KV capillary voltage, 30 V cone hole voltage, desolventizing gas nitrogen, 1000 l/h gas flow rate, 500°C desolventizing temperature, 150°C ion source temperature, 50 l/h inlet cone flow rate, 600 l/h desolventizing flow rate, scanning method = neutral gain scan mode. The mass spectrometry data acquisition and processing software was Mass Lynx V4.1.

### Antisense oligonucleotide gene-silencing experiments

To verify the possible function of *CsSDH4* and *CsSDH3* in catechin synthesis regulation in tea plants, the antisense oligonucleotide method [45] was employed to assess the expression levels of the two genes, respectively. Antisense oligonucleotide primers were designed using the soligo online website and diluted to a primer concentration of 20 μM (Table S9). The tea plant’s young spring shoots, comprising one bud and two leaves, were immersed in *CsSDH4* and *CsSDH3* antisense oligonucleotide primer solutions for 72 h, respectively. Sense oligonucleotide solution was used as a control. Subsequently, the tea samples were collected and immediately frozen in liquid nitrogen, and gene expression levels were analyzed by RT-qPCR, while the catechin contents were determined via HPLC.

### RT-qPCR analysis

Total RNA was extracted from the samples using an EASYspin Plus Complex Plant RNA Kit (Aidlab, Beijing, China). The cDNA was generated via reverse transcription reaction with a FastQuant RT Kit (Tiangen, Beijing, China). RT-qPCR analysis was performed on a Roche LightCycler 480 II Real-Time PCR system, utilizing the ChemoHS specificity Plus qPCR Mix (MonAmp, Suzhou, China). The 10-μl qPCR reaction mixture contained 5 μl of 2 × SYBR Green PCR master mix, 1 μl of cDNA (40 ng μl^−1^), 0.2 μl of each primer, and 3.6 μl of ddH_2_O. To ensure accuracy, *CsGAPDH* and *SIActin* were used as internal controls. The 2^-ΔΔCt^ method was used to calculate the relative fold change in gene expression. Primer sequences used in this study are shown in Table S9**.**

### Statistical analysis

For analysis of variance and multiple comparisons, we used SPSS version 16.0. Duncan’s multiple range test and Student’s *t*-test were used to identify significant differences at *P* < 0.05. The experimental results were calculated as the mean values ± standard deviation (SD) of three independent biological replicates. All figures and data results were generated and processed using Microsoft Excel 2019 or the online graphic production platform at https://www.bioinformatics.com.cn/.

## Supplementary Material

Web_Material_uhae356

## Data Availability

The raw sequencing data obtained in this study have been deposited into the NCBI Sequence Read Archive under the BioProject accession no. PRJNA1128726.
