# Supplementary material for: Two shikimate dehydrogenases play an essential role in the biosynthesis of galloylated catechins in tea plants
Source: Hortic Res. 2024 Dec 23;12(4):uhae356. doi: 10.1093/hr/uhae356 (PMC11891478; doi:10.1093/hr/uhae356)
Supplement: Web_Material_uhae356 [file web_material_uhae356.zip › Supplement figure.docx]

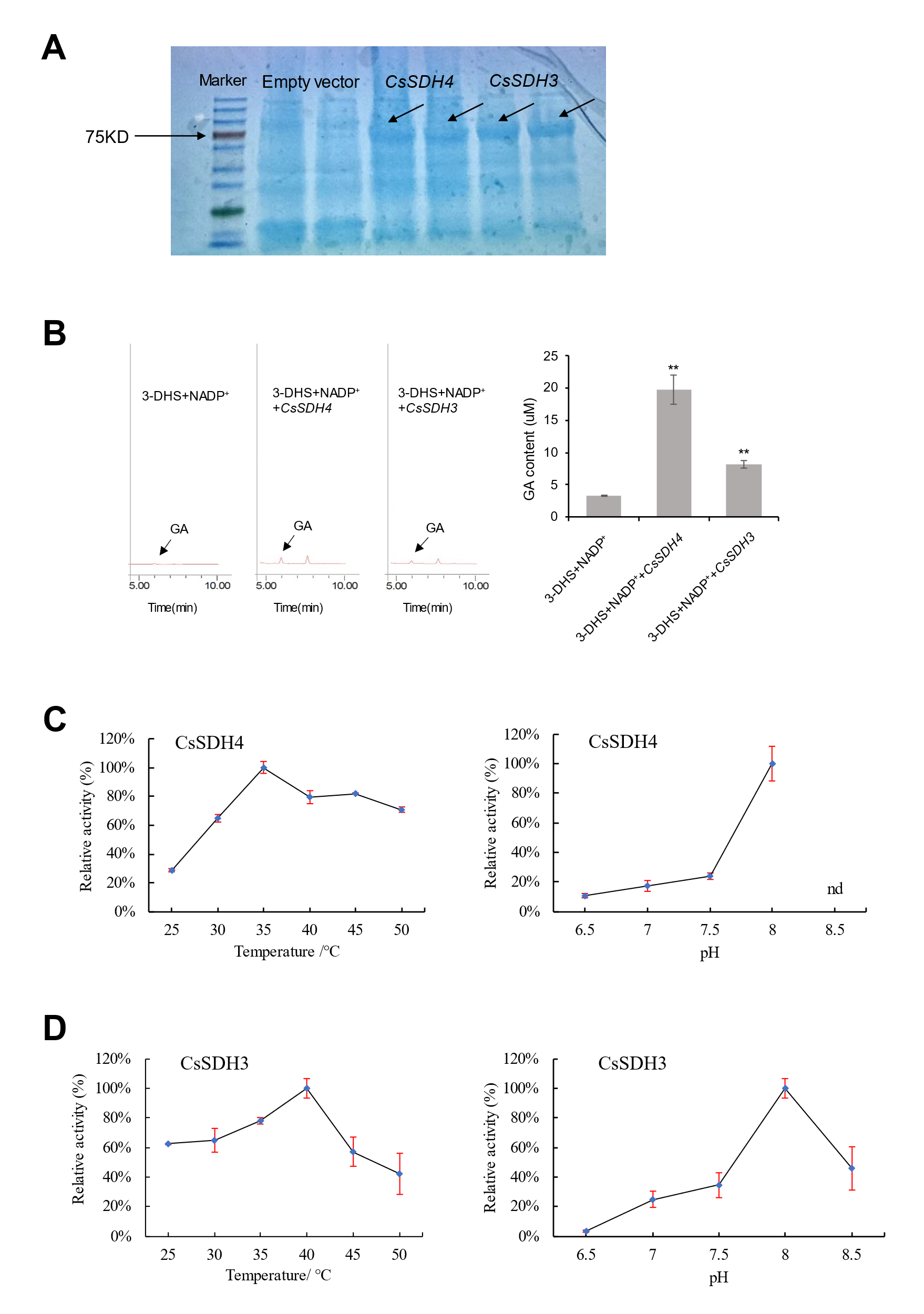


**Fig. S1: Optimum conditions for the GA generation capacity of CsSDH4, CsSDH3.**

(A) SDS-PAGE analysis of the crude enzyme of CsSDH4 and CsSDH3.

(B) The reaction products in the enzymatic assays were quantified by HPLC (λ=280nm).

(C) Effect of temperature and pH on enzyme activity of CsSDH4

(D) Effect of temperature and pH on enzyme activity of CsSDH3

Data are shown as the mean ± SD, and the three independent biological replicates, ** *p*<0.01 (Student’s t test), nd: not detected.


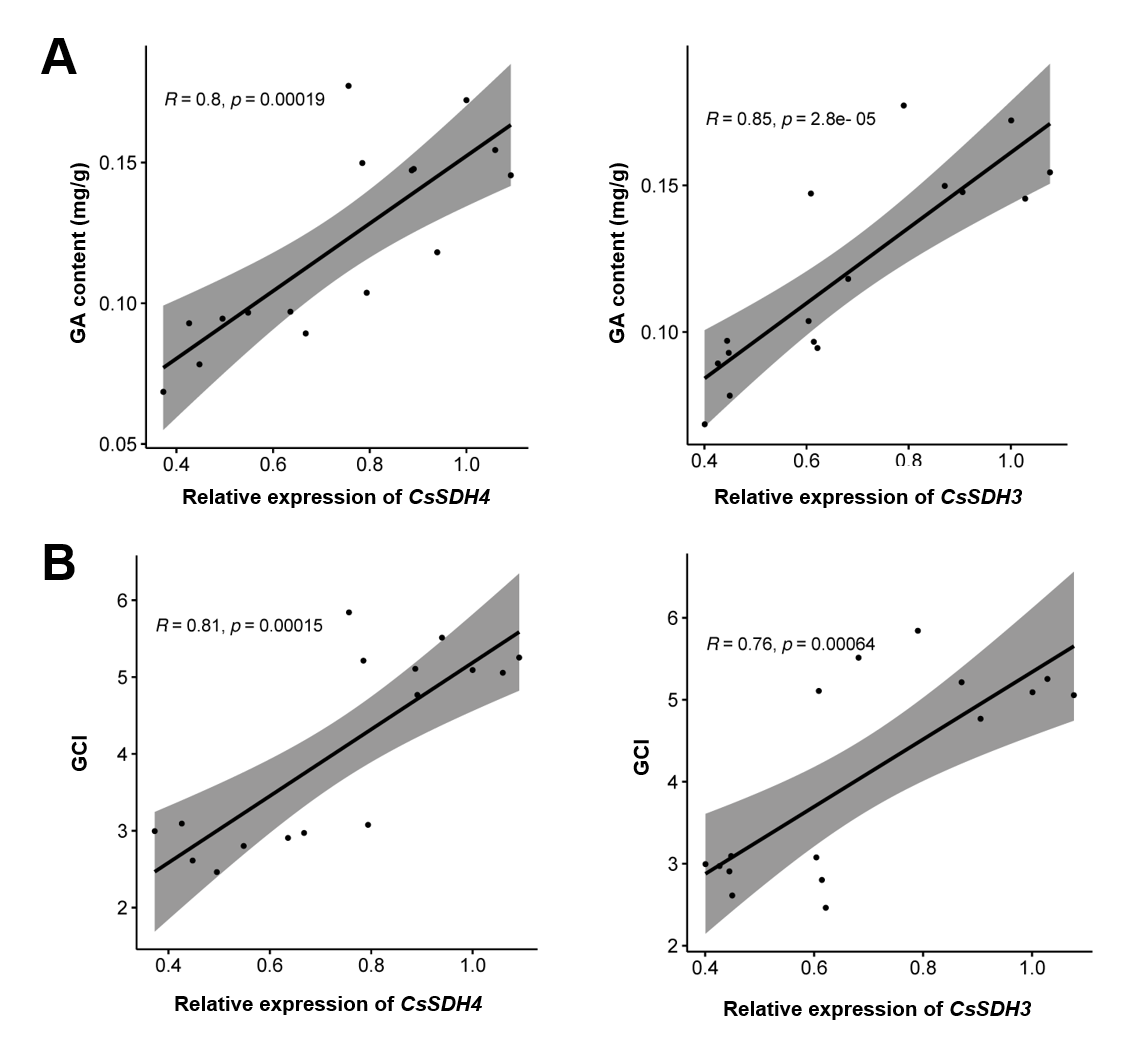


**Fig. S2: Correlation analysis of *CsSDH4*, *CsSDH3* with content of GA and GCI traits.**

(A) the GA content showed highly significant positive correlation with relative expression of *CsSDH4* (*R*=0.80, *P*=0.00019) and *CsSDH3* (*R*=0.85, *P*=2.8×10^-5^).

(B) the GCI traits showed highly significant positive correlation with relative expression of *CsSDH4* (*R*=0.81, *P*=0.00015) and *CsSDH3* (*R*=0.76, *P*=0.00064).


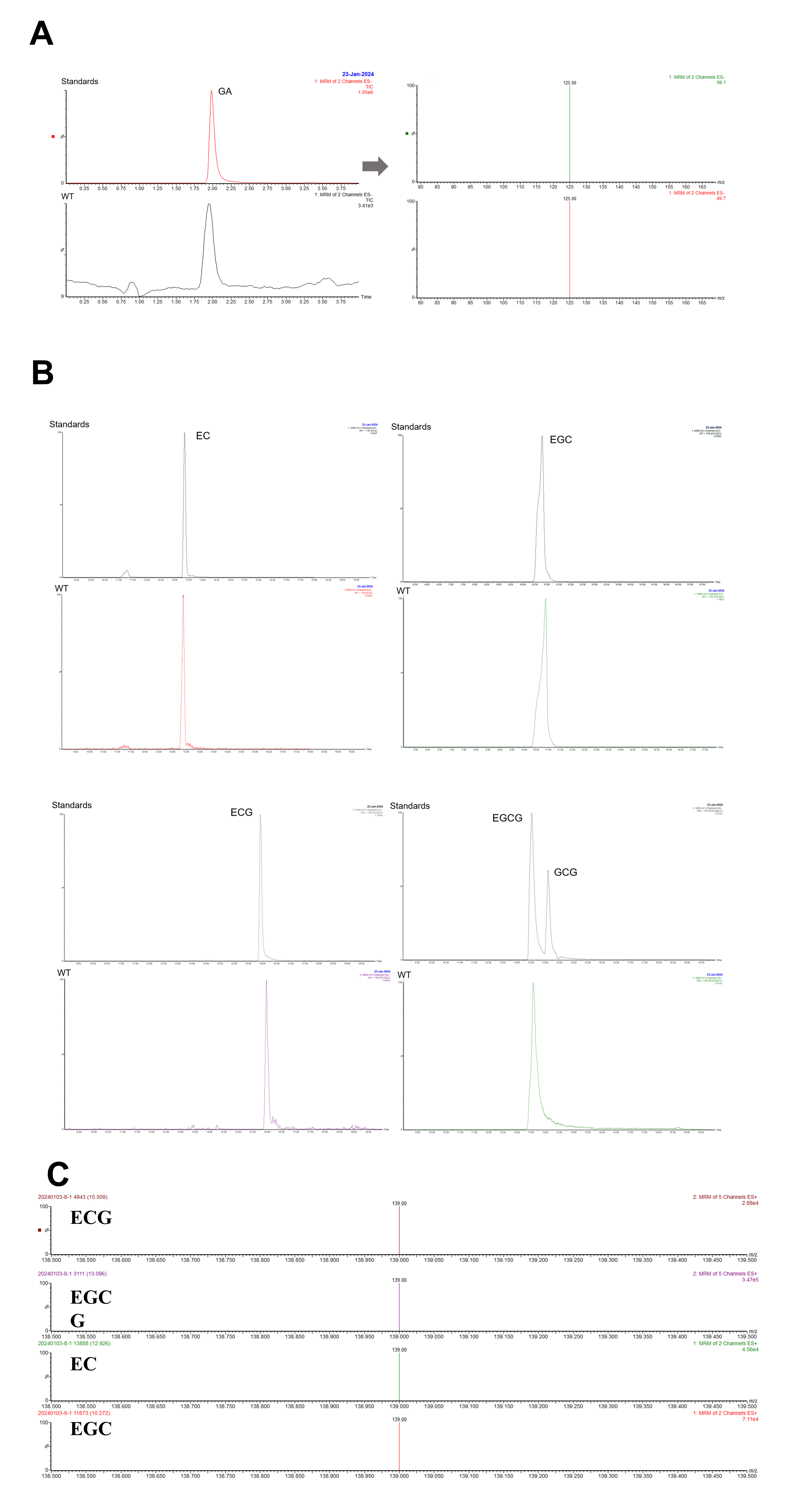


**Fig. S3: Identification of catechin fraction in tomato by LC-MS.**

(A) the content of GA is identified by LC-MS, the top of the picture is the GA content determined using the standard as control, and the bottom picture shows the GA content identified in tomato, the same as below, the GA with a retention time of 2.00 minutes corresponds to the protonated characteristic molecular ions of m/z 125.0.

(B) the content of EC, ECG, EGC, EGCG was identified by LC-MS.

(C) the mass spectrometry of EC, ECG, EGC, EGCG was identified by LC-MS, the ECG, EGCG, EC, EGC had the same protonated characteristic molecular ions of m/z 139.0 with different retention times (15.509min, 13.096min, 12.926min, 10.272min).


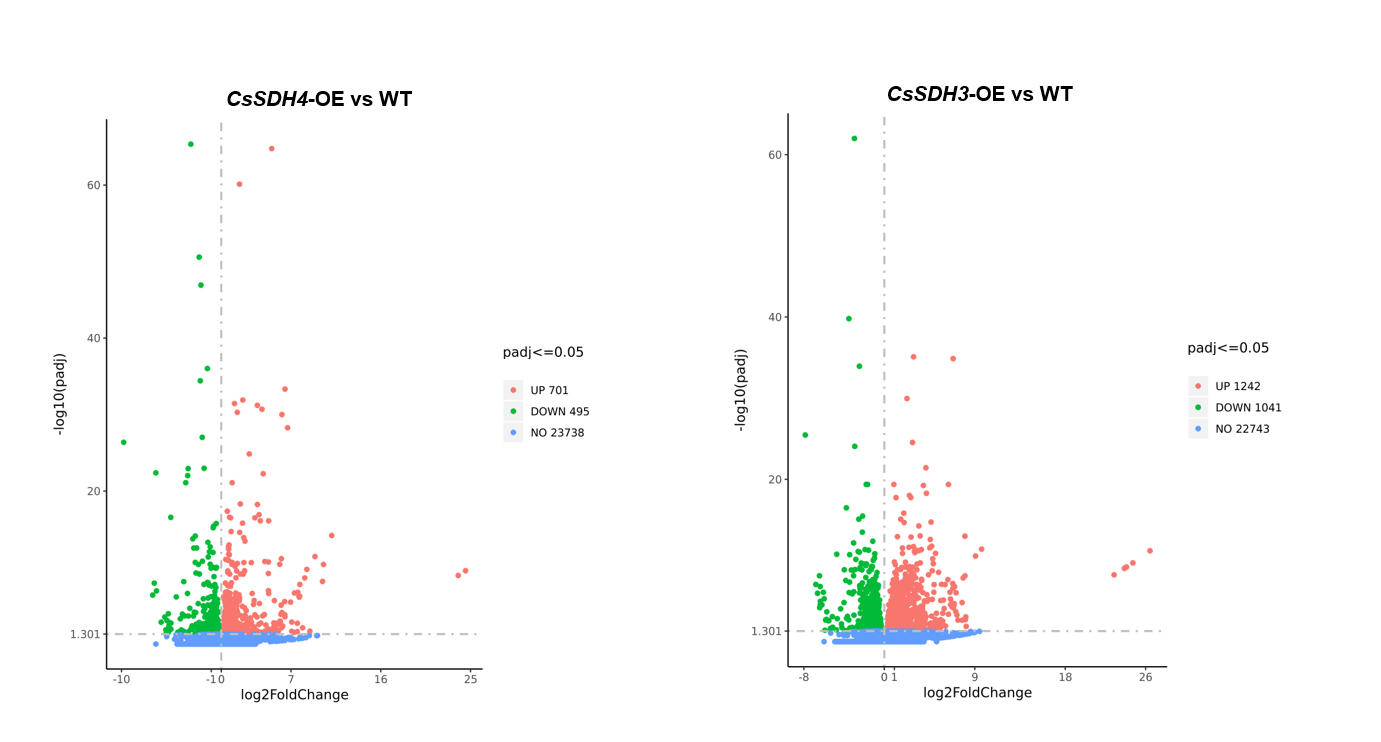


**Fig. S4: Differentially expressed genes in overexpression *CsSDH4* and *CsSDH3* compared to WT tomatoes by RNA-seq.**
